# The influence of affective states varying in motivational intensity on cognitive scope

**DOI:** 10.3389/fnint.2012.00073

**Published:** 2012-09-10

**Authors:** Eddie Harmon-Jones, Philip A. Gable, Tom F. Price

**Affiliations:** ^1^School of Psychology, University of New South WalesSydney, NSW, Australia; ^2^Department of Psychology, University of AlabamaTuscaloosa, AL, USA

**Keywords:** approach motivation, broadening, cognitive scope, EEG, emotion, ERP, narrowing, positive affect

## Abstract

We review a program of research that has suggested that affective states high in motivationally intensity (e.g., enthusiasm, disgust) narrow cognitive scope, whereas affective states low in motivationally intensity (e.g., joy, sadness) broaden cognitive scope. Further supporting this interpretation, indices of brain activations, derived from human electroencephalography, suggest that the motivational intensity of the affective state predicts the narrowing of cognitive scope. Finally, research suggests that the relationship between emotive intensity and cognitive scope is bi-directional, such that manipulated changes in cognitive scope influence early brain activations associated with emotive intensity. In the end, the review highlights how emotion can impair and improve certain cognitive processes.

We review research that has examined the effects of emotional states varying in motivational intensity on cognitive scope (narrowing/broadening). This research may yield insight into the issues with which this special issue is concerned. In particular, the research sheds light on the circumstances in which emotion may enhance vs. impair cognition and identifies individual differences that influence these effects.

Emotions (affective states) are complex psychophysiological constructs composed of many underlying dimensions. We define valence as the positive to negative evaluation of the subjectively experienced state (Harmon-Jones et al., 2011a,c). We define motivational intensity as the urge to move toward/away from a stimulus; it can range from low to high (Harmon-Jones et al., in press). Arousal, as measured subjectively and by activation of the sympathetic nervous system, is often posited to be a proxy for motivational intensity (Bradley and Lang, 2007). We concur with this definition but also posit that motivational intensity and arousal may be separable, as when one is aroused but not driven to engage in action (e.g., caffeine).

Our program of research has examined how affective states differing in motivational intensity influence the broadening or narrowing of cognition, which is defined as similar to cognitive expansiveness. It can occur at attentional or conceptual levels, it may involve the amount of information available in central executive resources (for comparing, maintaining, and manipulating) as well as perceptual resources (e.g., encoding), and it has been used in past discussions of the effects of emotions on cognition (e.g., Fredrickson, 2001). It has been measured in a number of ways, such as local/global attentional scope (Fredrickson and Branigan, 2005), visual selective attention (Rowe et al., 2007; Moriya and Nittono, 2011), perceptual encoding in the visual cortices (Schmitz et al., 2009), cognitive categorization (Isen and Daubman, 1984), and unusualness of associations (Isen et al., 1985).

As will be reviewed, affective states low in motivational intensity cause broadening, whereas affective states high in motivational intensity cause narrowing of cognitive scope. Thus, with regard to whether affective states impair or enhance cognition, we suggest on the basis of our research that certain affective states can enhance certain aspects of cognition while simultaneously impairing other aspects of cognition. Below, we explain our position in more detail.

## Positive affects vary in motivational intensity

Most previous research on the relationship between affective states and cognitive scope (broad vs. narrow) has examined positive affective states low in motivational intensity (amusement) and negative affective states high in motivational intensity (fear; Harmon-Jones and Gable, 2008). This confounding of affective valence with motivational intensity makes it difficult to claim that positive affect broadens (Fredrickson, 2001) and negative affect narrows cognitive scope (Easterbrook, 1959).

The prior research manipulated positive affect via gifts (Isen and Daubman, 1984), recall of past positive events (Gasper and Clore, 2002), or clips of humorous or satisfying events (Fredrickson and Branigan, 2005). We suggest that the positive affective state created by these manipulations is low in approach motivation because these manipulations create relatively passive states not associated with goal pursuit. Other positive affective states are higher in approach motivation, but they were not examined in past research on positive affect and cognitive scope.

This distinction between low and high approach motivated positive affect (which is on a continuum) is similar to other conceptualizations, such as ones that posit that appetitive or pre-goal positive states are different from consummatory or post-goal positive states (Knutson and Wimmer, 2007). Pre-goal and post-goal positive affect states are associated with different neural structures and neurochemicals (Panksepp, 1998; Knutson and Wimmer, 2007; Harmon-Jones et al., 2008).

We posit that pre-goal, high approach-motivated positive affective states, such as desire and enthusiasm, narrow cognitive scope, so that organisms are not distracted by peripheral details that may impede goal pursuit. In contrast, post-goal, low approach-motivated positive affective states, such as satisfaction, promote openness to new opportunities. After the goal is accomplished, a broad cognitive scope allows new goal opportunities to be identified and later pursued. Low approach-motivated negative affect, such as sadness, also broadens cognitive scope. When goals are terminally blocked and motivation lowers, broadened attention may assist in finding new solutions to the goal or finding a new goal.

## Attentional scope following low vs. high approach positive affect

In our program of research, we measured attentional scope using two commonly used measures of local/global processing. The first, the Kimchi and Palmer (1982) task, presents several trials. In each trial, three figures, each comprising three to nine local elements (triangles or squares), are presented. One figure, the standard, is positioned on top, and the two other figures, the comparisons, are positioned below. One of the comparison figures has local elements that matched the local elements of the standard, whereas the other comparison figure has a global element that matches the global element of the standard. Thus, judgments of which comparison figure are more similar to the standard figure are based on either the global element of one comparison figure or the local elements of the other comparison figure. Participants indicate their “first and most immediate impression” as to which of the two comparison figures in each triad best matches the standard figure, and their choice indicates whether they were more locally (narrowly) or globally (broadly) focused at the moment.

The second, the Navon (1977) letters task, also presents several trials. The stimuli in the letters task are large letters composed of smaller letters. Each vertical and horizontal line of a large letter is made up of five closely spaced local letters (e.g., an *H* made up of *F*s). Participants are asked to indicate “as quickly as possible” whether the picture contains the letter *T* or the letter *H*, by pressing one button for *T* and another button for *H*. Global targets are those in which a *T* or an *H* is composed of smaller *L*s or *F*s. Local targets are those in which a large *L* or *F* is composed of smaller *T*s or *H*s. Faster responses to the large than to the small letters indicate a global (broad) focus, whereas faster responses to the small than to the large letters indicates a local (narrow) focus.

To test the effects of low vs. high approach-motivated positive affect on attentional scope (using the Kimchi and Palmer, 1982, task), low approach positive affect was created with a film clip of funny cats, and high approach positive affect was created with a film clip of desserts (Gable and Harmon-Jones, 2008, Experiment 1). Self-report manipulation checks indicated that the appropriate positive affective states were evoked without any negative affect. In support of the hypothesis, the dessert film (which caused high approach positive affect) caused less broadening of attention than the funny cats film (which caused low approach positive affect).

Other experiments found that dessert pictures caused more narrowing of attention than neutral pictures (Gable and Harmon-Jones, 2008, Experiment 2). Also, individuals who scored higher in trait approach motivation showed even more narrowing of attention following appetitive pictures (Gable and Harmon-Jones, 2008, Experiment 3). Increased approach motivation caused by leading individuals to believe they would get to eat desserts following picture viewing evoked even more narrowing of attention (Gable and Harmon-Jones, 2008, Experiment 4). In addition, alcohol-related pictures cause narrowed attention for individuals who are motivated to consume alcohol (Hicks et al., 2012). The above experiments measured attentional scope with the Navon (1977) task. These positive affect manipulations increase self-reported positive affect (e.g., excited, enthusiastic) but not negative affect.

## Evoking low vs. high approach-motivated positive affect with money

Other experiments have tested the primary hypothesis by evoking positive affect using stimuli other than emotional pictures. Low vs. high approach positive affect was manipulated in one experiment using the monetary incentive delay paradigm (Knutson et al., 2000; Knutson and Wimmer, 2007). In this task, cues indicating the possibility of gaining money for task performance are used to evoke pre-goal (high approach) positive affect, and different cues indicating the outcome of the task performance (whether a reward was obtained) are used to evoke post-goal (low approach) positive affect.

In one experiment (Gable and Harmon-Jones, 2010a), cognitive scope was measured by assessing recognition memory for neutral words that were presented either in the center of the computer monitor or in the periphery of the computer monitor. We found that superior memory for centrally presented words after pre-goal positive affect cues than after post-goal positive affect cues. In contrast, memory for peripherally presented words was superior after post-goal positive affect cues than pre-goal positive affect cues. Two experiments have conceptually replicated these results with Navon's (1977) local/global attentional scope task (Gable and Harmon-Jones, 2011a).

## Perceptual vs. conceptual processing following low vs. high approach positive affect

To test whether low vs. high approach positive affect would influence other, more conceptual cognitive processes, we conducted two experiments in which narrowing/broadening of cognition was measured using Isen and Daubman's (1984) cognitive categorization task (Price and Harmon-Jones, 2010). In addition, we manipulated low and high approach positive states with an embodiment manipulation (Harmon-Jones et al., 2011b). In the high approach positive affect condition, participants smiled and leaned forward in a chair, similar to how one might lean toward an object of desire. In the low approach positive affect condition, participants smiled and reclined backward in a reclining chair, similar to how one might recline after goal accomplishment. In a moderate approach positive affect condition, participants sat upright and smiled. In the categorization task, which was completed while participants were in one of these postures, participants rated the extent to which weakly associated exemplars (e.g., camel) of a particular category (e.g., vehicle) fit within that category. The high approach positive condition produced the most narrow categorizations (i.e., participants were more likely to indicate that the exemplars did not belong to the category), followed by the moderate approach positive condition, and then the low approach positive condition.

## Neuroscientific evidence

To examine neural processes underlying the effects of approach positive affect on cognitive narrowing, we have conducted experiments using measures of electrical brain activity. In one experiment (Harmon-Jones and Gable, 2009), we measured electroencephalographic (EEG) alpha power to neutral and dessert picture primes and measured attentional scope following each prime using the Navon (1977) letters task. We focused on asymmetric frontal cortical activity, because greater relative left frontal cortical activity has been found to relate to approach motivation (Coan and Allen, 2004; Harmon-Jones et al., 2010). Results indicated that greater relative left frontal activity to the dessert pictures (but not neutral pictures) predicted attentional narrowing immediately following the dessert picture primes (Harmon-Jones and Gable, 2009).

In another experiment, we used the same methods (affective pictures and Navon letters task) but examined event-related brain potentials (ERP), specifically the late positive potential (LPP) of the ERP (Gable and Harmon-Jones, 2010b), which is sensitive to the motivational significance of stimuli (for review, see Hajcak et al., 2012). We found that the LPP was larger to dessert than neutral pictures. This LPP effect occurred over several brain regions, including medial central and parietal cortices. It also showed an asymmetric effect over the frontal cortex, with the dessert pictures evoking larger LPPs over the left than right frontal cortex. Importantly, LPPs to dessert pictures predicted attentional narrowing following the dessert pictures (no significant correlations were observed for neutral stimuli).

## Comparing negative affective states differing in motivational intensity

Based on the reviewed research, we suggest that the motivational intensity of the positive affect determines whether positive affect causes broadening or narrowing of cognitive scope. Does the motivational intensity of the negative affective state influence cognitive scope? Some past research on depression suggested that low intensity negative affect causes broadening (von Hecker and Meiser, 2005). We examined whether negative affects varying in motivational intensity influence cognitive scope (Gable and Harmon-Jones, 2010c). Low motivationally intense negative affect was created with pictures of sad events, whereas high motivationally intense negative affect was created with pictures of disgusting events. Both types of pictures evoked greater self-reported negative affect than neutral pictures did. However, sad pictures evoked lower self-reported arousal than disgust pictures. This suggests that the disgust pictures evoked greater motivational intensity than the sadness pictures. Conceptually consistent with the positive affect results, sad pictures broadened attention, whereas disgust pictures narrowed attention relative to neutral pictures (measured with the Navon, 1977, task).

In contrast to the above results, past studies suggest different outcomes for sadness. Gasper and Clore (2002) had participants write about “a personal life event that had made them feel either ‘happy and positive’ or ‘sad and negative’.” In Study 1, the manipulation check was similarly worded. In Study 2, the same manipulation was used and negative affect was measured with a wide array of negative emotion words. Gasper and Clore averaged all of these words together, suggesting that the affective state manipulated was a mix of negative states and not sadness alone. Rowe et al. (2007) found no differences between a neutral state and a sad state on attentional narrowing. We suggest that the effect of sadness on attentional scope may depend on whether the sadness evoked is lower or higher in motivational intensity; the latter may occur when sadness is mixed with other negative affects. Research is needed to test this idea.

These results suggest the need for a concept that explains how certain positive (e.g., desire) *and* negative (e.g., disgust) affects cause narrowing, whereas other positive (e.g., amusement) *and* negative (e.g., sadness) affects cause broadening. We suggest that the concept of motivational intensity explains these effects: low motivationally intense affects broaden cognitive scope whereas high motivationally intense affects narrow cognitive scope.

## Affective states, arousal, and motivational intensity

Does arousal rather than motivational intensity better explain how these different affective states influence cognitive scope? If arousal is the same as motivational intensity, as some have posited (Bradley and Lang, 2007), then the arousal explanation is the same as the motivational intensity explanation.

On the other hand, arousal and motivational intensity may occasionally be separate constructs. Humor evokes an arousing state that is low in approach motivational intensity: humor does not urge action toward something. Humorous films cause more attentional broadening than neutral films (Fredrickson and Branigan, 2005; Gable and Harmon-Jones, 2008), thus suggesting that arousal per se cannot account for the effect of high approach positive affective states on attentional narrowing.

We recently tested another instance where arousal and motivational intensity are separable. That is, arousal can be prompted by physical exercise but this state is not necessarily associated with motivational intensity (Gable and Harmon-Jones, under review). In this experiment, participants were randomly assigned to pedal a stationary bike or not while performing the appetitive vs. neutral picture/attentional scope task (Navon, 1977). Individuals who pedaled had faster heart rates than individuals who did not. More importantly, manipulated arousal had no effect on attentional scope. These results suggest that motivational intensity, rather than arousal *per se*, causes attentional narrowing.

## The effect of cognitive scope on motivational intensity

Several experiments have revealed that affective states differing in motivational intensity influence attentional scope. Does attentional scope influence motivational intensity? Focusing narrowly on a motivationally significant object may increase motivation for the object, whereas considering the same object from a broader perspective may decrease motivation for the object.

We have tested this hypothesis in two experiments with appetitive and aversive stimuli. In these tests, we examined motivational processing by measuring ERPs to appetitive and neutral pictures. We focused on the N1 component, an early ERP component related to selective attention that is larger to motivationally significant stimuli (Keil et al., 2001; Foti et al., 2009).

Immediately prior to the presentation of each affective or neutral picture, attentional scope was manipulated by having participants simply indicate what letter was displayed at the local or global level in a between-subjects design. That is, participants viewed Navon (1977) letters and indicated the letter that was displayed as a local element or they indicated the letter that was displayed in the global configuration.

As compared to a global attentional scope, a local attentional scope caused larger N1 amplitudes to appetitive (Gable and Harmon-Jones, 2011b) and aversive pictures (Gable and Harmon-Jones, under review) but not to neutral pictures. These results suggest that the relationship between narrowed attentional scope and motivational intensity is bi-directional. Other work suggests that priming a local vs. global attentional scope can influence how positive and negative moods (with unknown levels of motivational intensity) influence attentional scope (Huntsinger et al., 2010).

## Conclusion

The evidence we reviewed suggests a revision to the well-accepted idea that positive affect broadens and negative affect narrows the scope of cognition. The reviewed evidence is consistent with previous evidence but suggests that previous results likely occurred because affective valence was confounded with motivational intensity: low motivationally intense positive affects were compared with high motivationally intense negative affects. The reviewed research manipulated affective valence independently of motivational intensity, and found that affective states low in motivational intensity broaden and affective states high in motivational intensity narrow the scope of cognition. Further research is needed to investigate the role of specific neural regions (e.g., amygdala, nucleus accumbens) and neurochemical processes (e.g., dopamine, opioids) within these regions in the effect of emotive states on attentional scope.

We suspect that motivationally intense affective states cause narrowing of cognitive scope because this often facilitates adaptive behavior that results in goal accomplishment (approach or avoidance of the stimulus). However, the narrowed cognitive scope that occurs with high motivationally intense affective states may hinder perception and processing of peripheral (or global) information that would prove useful. On the other hand, low motivationally intense affective states broaden cognitive scope, which may allow new goal opportunities to be identified and later pursued. However, the broadened scope that occurs with low motivationally intense affective states may hinder perception and processing of central (or local) information that would prove useful. Together, this body of research suggests that emotion may impair and improve cognitive processes depending on the situation in which the emotion occurs.

### Conflict of interest statement

The authors declare that the research was conducted in the absence of any commercial or financial relationships that could be construed as a potential conflict of interest.
